# Identifying late Pleistocene and Holocene refugia for baboons

**DOI:** 10.1038/s42003-025-08419-8

**Published:** 2025-07-04

**Authors:** James Blinkhorn, Dietmar Zinner, Lucy Timbrell, Andrea Manica, Matt Grove, Eleanor M. L. Scerri

**Affiliations:** 1 Department of Archaeology, Classics, and Egyptology, University of Liverpool, Liverpool, UK; 2 Human Palaeosystems Group, Max Planck Institute of Geoanthropology, Jena, Germany; 3 Cognitive Ethology Laboratory, German Primate Center, Leibniz Institute for Primate Research, Göttingen, Germany; 4 Department of Primate Cognition, Georg-August-Universität Göttingen, Göttingen, Germany; 5 Leibniz ScienceCampus Primate Cognition, Göttingen, Germany; 6 Department of Zoology, University of Cambridge, Cambridge, United Kingdom; 7 Department of Classics and Archaeology, University of Malta, Faculty of Arts, Msida, Malta; 8 Institute of Prehistoric Archaeology, University of Cologne, Cologne, Germany

**Keywords:** Ecological modelling, Palaeoecology

## Abstract

Climate change has the scope to significantly modulate the distribution of floral and faunal taxa, with those regions persistently suitable to a population through the largest environmental perturbations termed “refugia”. Within Africa, focus has been placed on forest refugia during glacial cycles as hotspots of biodiversity, whilst refugia for savannah species have been overlooked. We compiled a comprehensive dataset of baboon occurrences and fitted species distribution model ensembles to predict the present potential habitable range of each species and the genus as a whole. We then hindcasted these models to palaeoclimate reconstructions spanning the Late Pleistocene and Holocene in 1-thousand-year time steps to predict potentially habitable ranges throughout a full interglacial-glacial cycle. Our results indicate a substantial mosaic of refugia in the eastern African Rift Valley system, a discrete refugium in southern and south-western Africa, as well as isolated refugia across western Africa and Arabia. Orbital precession and obliquity both play a role in driving maxima and minima or predicted habitable ranges for alternate baboon species, but these appear expressed within ca. 100 thousand-year eccentricity cycles. This supports the use of full interglacial-glacial cycles, rather than simply comparing peak glacial and interglacial conditions, to determine the presence of refugia.

## Abstract (web summary)

Hindcasted species distribution modelling of baboons illuminates potential refugia across Africa and Arabia, with predicted maxima and minima of habitable ranges pulsed by orbital precession and obliquity expressed within the last precessional cycle.

## Introduction

Climate change plays a major role in modulating the geographic distribution of a wide range of taxa, for example by shifting habitats, modifying the suitability of habitats and opening and closing dispersal corridors between them^1^. In this context, and given a certain degree of ecological conservatism, refugia are of particular importance^2^. Refugia are regions that are persistently habitable to a given species or population throughout major cycles of environmental perturbation^3–6^. Early studies of refugia focused on the expansion of tree species from their most restricted distributions during the Last Glacial Maximum (LGM) to the more widespread distribution observable in the present day^7,8^. One hundred thousand-year eccentricity-driven glacial-interglacial cycles (or more typically glacial maxima and post-glacial responses) have been tacitly adopted as the timeframe to identify refugia^9–11^. Recent studies stress the role of ~23 thousand-year precession cycles to force high-latitude glaciation^12^, with precession having stronger influences on summer insolation at lower latitudes that may impact patterning in range expansion and contraction^13^. Notably, legacies of Quaternary palaeoclimatic change are widely evident in studies of modern tropical mammal diversity^14^, highlighting the broad significance of identifying refugia. Refugia models are routinely used as one means to explain patterns of geographically structured alpha diversity as well as intraspecific genetic diversity^15,16^. For example, individuals with shared geographic origin from the same refugium are more likely to be genetically similar to one another than individuals with origins in alternate, remote refugia, regardless of present geographic proximity. In such cases, refugial ancestry better explains patterns of genetic diversity of populations than if genetic diversity were structured by geographic proximity (i.e., isolation by distance), though both may be confounded by allopatry (e.g., for African apes^17^).

**Fig. 1:**
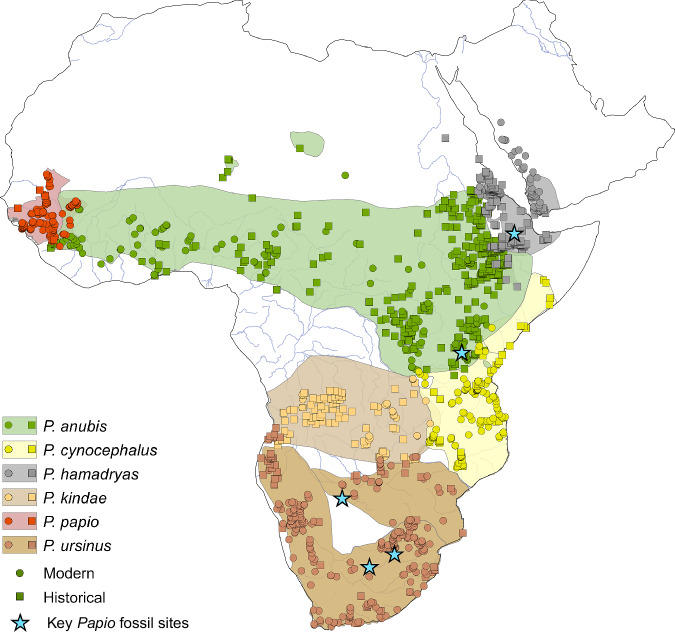
Map of modern and historical distribution of baboons with key fossil localities. Map illustrating modern (circles) and historical (squares) presence points of each modern baboon species synthesised for this study (Supplementary Information) with the presence of key fossil baboon specimens^35^ (stars), over the present species ranges of each baboon species^76–81^ in continental Africa and Arabia. Map created using ArcMAP 10.5 and made with Natural Earth.

Primates, like most other taxa, face considerable impacts from both current climate change and more direct human activity, such that the identification of primate refugia may be important to mitigate conservation issues in the future^18^. Substantial focus has been placed on the identification of African forest refugia, broadly coinciding with the LGM^19–23^, which have been shown to be important for a number of African primate taxa^17,20,24^. African refugia within more open savannah habitats are comparatively understudied^25^, with potential for greater dynamism in such habitats as they are constrained by the expansion and contraction of both forests and deserts. Baboons are presently widespread in savannah habitats across Africa and are able to adapt to a broad range of environmental conditions^26–28^. Recent phylogenetic studies across the six baboon species demonstrate complex patterns of genetic population structure, suggesting a Southern African origin of the genus in the Middle Pleistocene and a successive range expansion into Eastern Africa and along the northern savannah belt into Western Africa^29,30^. This movement was accompanied by a long history of interspecific gene flow due to temporary isolation and reconnection of populations^30–33^. In particular, the distribution of baboon mitochondrial haplotypes over the African landscape indicates a strong geographical pattern^29,33,34^ that might partly originate from periods of population isolation within refugia, requiring the identification of species-specific refugia for testing. An examination of Late Pleistocene/Holocene refugia based on contemporaneous occurrences is not currently possible, due to the sparse nature of the fossil record in time^35^ (Fig. 1). Moreover, the biased distribution of fossil preservation is likely to significantly underestimate past spatial diversity^36^. As a result, any examination of baboon refugia must be based on modern distribution patterns and projected to the past.

The combination of geographic range expansion, ecological flexibility alongside adaptations to savannah habitats, and complex sociality have led to baboons being used as an analogy for early hominin populations^37–40^. This includes the use of modern baboons as analogues for early hominins, such as australopithecines, with the fossil record demonstrating comparable patterns of evolution and radiation within shared landscapes and habitats over similar timeframes^41^. More recent studies extend such analogies to larger-bodied hominins, including models of social evolution amongst *Homo erectus*^42^ and morphological changes associated with hybridisation between closely related species such as *Homo sapiens* and Neanderthals^43^. Determining the present distribution of baboons and modelling potential distributions in the past provides a suitable analogy to evaluate how patterns of climate change may have modulated the availability of savannah habitats, identified as suitable for both baboons and hominin populations, which helps to avoid problems of spatial bias in the fossil record^36^. Additionally, evidence for expansions between Africa and Arabia is likely to be particularly pertinent for understanding human expansions out of Africa^44–46^. Moreover, identifying baboon refugia offers the potential to examine how climate-driven population isolation and connection at a continental scale may explain genomic patterning, with implications for understanding the generation of population structure amongst both earlier and later hominins^29,31,32,47–49^.

Here, we model the current distribution of suitable habitats for baboon species across Africa and Arabia using species distribution modelling approaches and extrapolate this to illuminate the past predicted habitable range of baboons throughout the Late Pleistocene and Holocene by identifying the modelled distribution of suitable habitats. We then identify and describe those landscapes that are modelled to have been persistently habitable to the genus and each species, examining how their refugia relate to changing extents of the wider predicted habitable ranges through time. Finally, we explore overlaps in predicted habitable ranges between baboon species through time as hybridisation at species boundaries is a common phenomenon in *Papio*^32^, and areas of overlap of suitable habitat most likely represent areas where gene flow among species occurred.

## Results

We compiled a dataset from both modern (*n* = 777) and recent historical (*n* = 625) reports of baboon presence, recording the latitude, longitude, and species (Fig. 1; Supplementary Data 1). We employed this dataset in an ensemble species distribution modelling approach using the *tidysdm* R package^50^ using modern climatic, ecological, and geographic data from a statistical emulator of the HadCM3 global circulation model^51^ at a 30-arc-minute resolution accessed through the *pastclim* R package^52^ (see Methods). Additional analyses at higher spatial resolutions (10-arc minutes) did not substantially alter the results and support our analyses at 30-arc-minute resolution (Supplementary Information). We selected predictor variables independently for each species, with the resulting models yielding averaged AUC values ranging from 0.8 to 0.94, indicating good model accuracy (Table 1), with limited differences from using a shared suite of predictor variables based on the distribution of the genus in preliminary testing.

**Table 1:** Mean area under the curve (AUC) and true skill statistic (TSS), and variable importance for each model ensemble, calculated as 1-AUC loss after 10 permutations, for each baboon species and the genus

	*P. anubis*	*P. cynocephalus*	*P. hamadryas*	*P. kindae*	P. papio	*P. ursinus*	*Papio*
Mean Boyce continuous index	0.878	0.768	0.796	0.752	0.624	0.742	0.922
Mean AUC	0.817	0.802	0.935	0.826	0.853	0.816	0.823
Mean TSS	0.510	0.544	0.787	0.600	0.689	0.546	0.510
Annual mean temperature (Bio01)	0.077	-	-	-	-	-	0.075
Temperature seasonality (Bio04)	-	-	0.048	0.188	-	-	-
Max temperature of warmest month (Bio05)	-	-	-	0.073	-	-	-
Temperature annual range (Bio07)	-	0.080	-	-	-	-	-
Mean temperature of wettest quarter (Bio08)	-	-	0.032	-	-	-	0.066
Mean temperature of the driest quarter (Bio09)	-	0.116	0.032	-	-	0.093	0.069
Mean Temperature of Coldest Quarter (Bio11)	-	-	-	-	0.101	-	-
Mean annual precipitation (Bio12)	-	-	-	-	-	0.234	-
Precipitation of wettest month (Bio13)	0.176	0.075	-	-	-	-	0.224
Precipitation of driest month (Bio14)	0.057	-	0.038	-	0.126	-	0.050
Precipitation seasonality (coefficient of variation)(Bio15)	0.078	0.063	-	-	0.121	-	0.077
Precipitation of wettest quarter (Bio16)	-	-	-	0.131	-	-	-
Precipitation of driest quarter (Bio17)	-	-	-	-	-	0.084	-
Precipitation of warmest quarter (Bio18)	-	0.077	-	0.084	-	-	-
Precipitation of coldest quarter (Bio19)	0.091	0.127	0.073	0.199	0.042	-	0.053
Rugosity	0.113	0.067	0.207	-	0.093	0.219	0.202

We employed these modelled present distributions to predict past potential habitable ranges of each baboon species for each 1000-year timestep spanning the Late Pleistocene and Holocene (130-0 thousand years ago [ka]), as binary presence/absence time-slices. We employed two alternative methods to constrain the predicted habitable range of each species through time and to identify potential refugia (see Methods). The more conservative *Step-Wise* approach first identified and retained patches of modelled predicted habitable range in the present day that overlap with the presence dataset, and subsequently retained patches where overlap is identified with retained patches in the preceding time-slice in a step-wise fashion from 1 to 130 ka. The simpler *Summed* approach combined all modelled time-slices and retained patches of potential range where overlap with the presence dataset is identified for at least one time-slice. In each case, potential refugia were identified where a given cell was included as part of the predicted habitable range in every time slice. The absence of direct overlaps in the predicted habitable range between at least one pair of adjacent time-slices prohibited the use of the *Step-Wise* approach for *P. kindae*; both methods were applicable in all other cases. Changes in the Step-Wise, Summed, and full predicted habitable range through time for each baboon species and the whole genus are presented as Supplementary Movies 1–7.

Substantial differences between species occur in the scale of the predicted habitable range and potential refugia that are predicted, broadly consistent with patterns evident in their present-day distributions (Table 2). The predicted habitable range of *P. anubis* throughout the Late Pleistocene is larger than that of other species and comparable in scale to the predicted habitable range of the genus as a whole, with the predicted habitable range of *P. hamadryas* and *P. ursinus* consistently smaller than other species, with both patterns consistent across the two approaches. Both the scale and proportion of potential refugia identified are broadly comparable between models, ranging between 0 and 27% of the predicted habitable range. Potential refugia for *P. cynocephalus* and *P. papio* are notably smaller than other species, with no refugium predicted for *P. kindae*, indicating the limited geographic stability of their predicted habitable range throughout the Late Pleistocene and Holocene.

**Table 2:** Area of predicted habitable range and potential refugia, and the proportion of predicted habitable range identified as refugia across baboon species and genus, differentiating *Step-Wise* and *Summed* approaches

Approach	Variable	*P. anubis*	*P. cynocephalus*	*P. hamadryas*	*P. kindae*	*P. papio*	*P. ursinus*	*Papio*
Summed	Potential Refugia (1000 km^2^)	2926	65	444	0	31	591	5326
	Potential Range (1000 km^2^)	16541	13541	1848	8696	9596	3345	19649
	Proportional Refugia %	17.7	0.5	24	0	0.3	17.7	27.1
Step-wise	Potential Refugia (1000 km^2^)	2255	6	367	-	9	591	5212
	Potential Range (1000 km^2^)	16073	13153	1848	-	8514	3250	19267
	Proportional Refugia %	14	<0.1	19.8	-	0.1	18.2	27.1

**Fig. 2:**
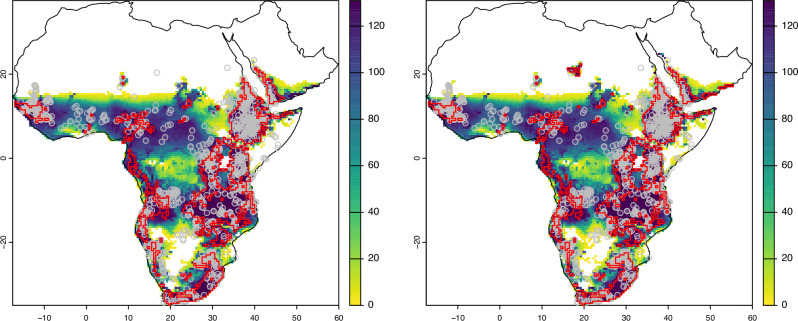
Maps illustrating predicted habitable ranges and refugia for *Papio*. Predicted Step-Wise (left) and Summed (right) habitable range for the genus *Papio*, with the colour scale illustrating the number of time-slices cells that are predicted to form part of the habitable range, with potential refugia (where cell count = 131) outlined in red; modern and recent historical presence data are illustrated as grey circles.

**Fig. 3:**
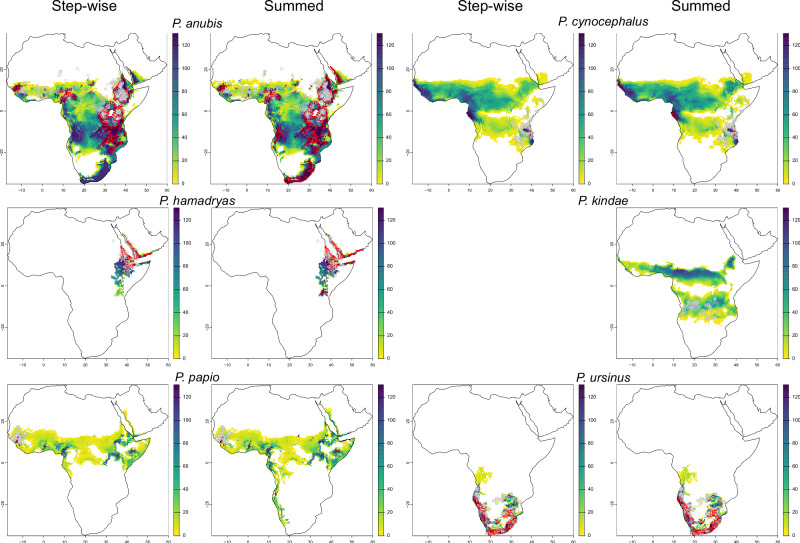
Maps illustrating predicted habitable ranges and refugia for each baboon species. Predicted step-wise and summed habitable range for each baboon species, with the colour scale illustrating the count of time-slices cells that are predicted to form part of the habitable range, with potential refugia (where cell count = 131) outlined in red; modern and recent historical presence data are illustrated as grey circles. No step-wise continuity in the predicted habitable range was identified for *P. kindae*.

The distribution of predicted habitable range and refugia for baboon species throughout the Late Pleistocene and Holocene are illustrated in Figs. 2 and 3. Considerable overlap is identified between the two methods for the *Papio* genus (Fig. 2), with the predicted habitable range spanning much of Sub-Saharan Africa and including the western and southern margins of Arabia. A large potential refugium spanning the eastern African Rift System is identified, with a smaller, elongated potential refugium spanning the south and central-western African coast, and smaller, more isolated potential refugia in the Yemeni, Cameroonian and Guinean highlands. A broadly similar pattern is observed for *P. anubis* potential refugia, though with a more fragmented presence in southern Africa. Predicted habitable range and refugia for *P. hamadryas* and *P. ursinus* closely correlate to their present distribution (Fig. 3). Considerable overlap in the predicted habitable range across much of tropical Africa is observed for *P. cynocephalus* and *P. kindae*, with potential refugia identified in West Africa, closely corresponding to the present distribution and potential refugia of *P. papio* (Fig. 3).

**Fig. 4:**
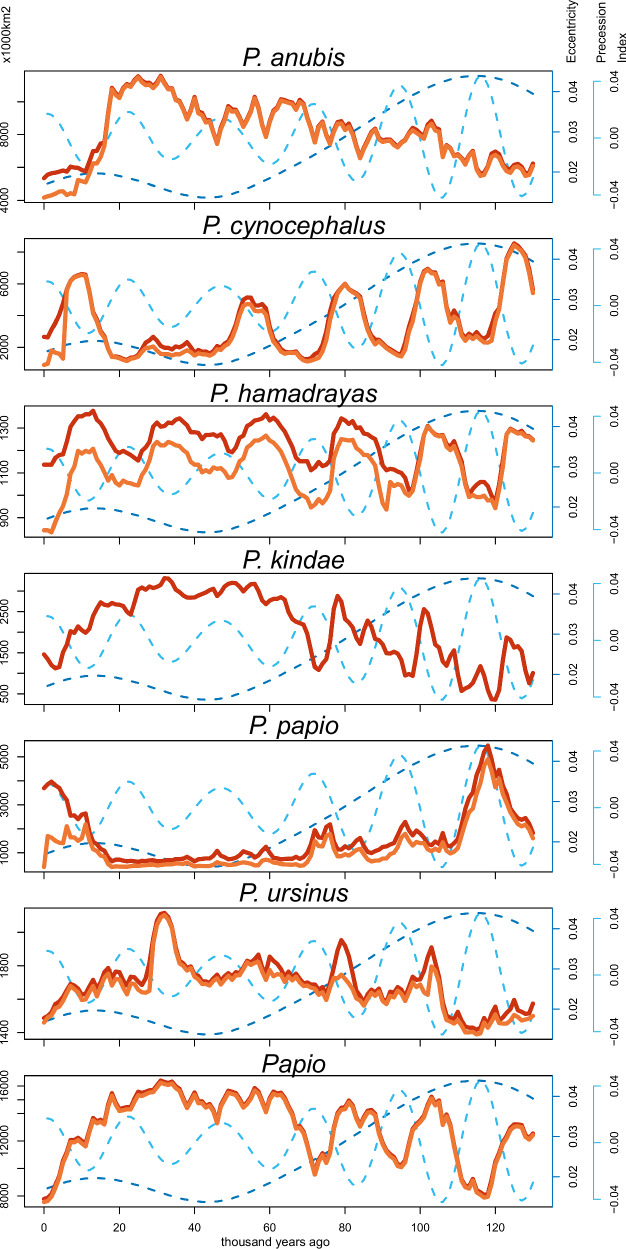
Graphs illustrating extent of predicted habitable range for each species and the genus Papio through the Late Pleistocene and Holocene. Area (1000 km^2^) identified within predicted habitable range in the Late Pleistocene and Holocene for each species using step-wise (orange) and summed (red) approaches, with respect to eccentricity (dashed dark blue) and precession (dashed light blue)^54^.

Substantial fluctuations in the predicted habitable range of baboon species and the genus as a whole are evident through time (Fig. 4), with seasonal variability in precipitation and temperature, rather than annual variability, playing more important roles in the models for all species except for *P. ursinus* (Table 2). Orbitally driven climate change may reflect patterning in overlapping cycles of eccentricity, precession, and obliquity, as well as how they interact to determine annual insolation, with eccentricity-modulated precession considered dominant in African tropical environments with substantial effects on seasonality and monsoonal intensity^53^. We conducted linear regressions between the predicted extent of the Summed range for each baboon species and these orbital parameters^54^, with the results shown in Table 3. All baboon species and the genus as a whole show a significant relationship to changes in eccentricity, featuring ca. 100 kyr cycles, with positive relationships seen for *P. cynocephalus* and *P. papio*, whereas negative relationships are observed for all other species and the *Papio* genus. Obliquity, with ca. 41kyr cycles, shows a significant, negative relationship in the predicted habitable range of *P. anubis*, and a positive relationship to the predicted habitable range of *P. cynocephalus* and *P. hamadryas*. The predicted habitable range of the *Papio* genus, and that of all species except for *P. anubis*, shows a significant relationship to climatic precession, with a negative relationship observed apart from for *P. papio*. Significant relationships between predicted habitable ranges with mean annual insolation were only observed for two species, with a positive relationship identified for *P. hamadryas* but a negative relationship seen with *P. papio*.

**Table 3:** Results of regression between the extent of the predicted habitable range of each baboon species through time and orbital parameters that influence climate change

Species	Statistic	Eccentricity	Climatic precession	Obliquity	Insolation
***P. anubis***	*R*^2^	0.293	0.002	0.141	0.000
	*F*	53.485	0.323	21.221	0.047
	*p*	0.000	0.571	0.000	0.829
***P. cynocephalus***	*R*^2^	0.181	0.417	0.109	0.005
	*F*	28.494	92.226	15.833	0.619
	*p*	0.000	0.000	0.000	0.433
***P. hamadryas***	*R*^2^	0.217	0.593	0.046	0.136
	*F*	35.846	188.099	6.156	20.249
	*p*	0.000	0.000	0.014	0.000
***P. kindae***	*R*^2^	0.597	0.050	0.003	0.003
	*F*	191.474	6.770	0.370	0.405
	*p*	0.000	0.010	0.544	0.526
***P. papio***	*R*^2^	0.278	0.074	0.001	0.134
	*F*	49.561	10.297	0.155	19.943
	*p*	0.000	0.002	0.694	0.000
***P. ursinus***	*R*^2^	0.340	0.058	0.014	0.008
	*F*	66.586	8.010	1.798	1.092
	*p*	0.000	0.005	0.182	0.298
***Papio***	*R*^2^	0.340	0.058	0.014	0.008
	*F*	66.586	8.010	1.798	1.092
	*p*	0.000	0.005	0.182	0.298

Besides identifying these relationships to orbital parameters, some broader patterns in changes in the predicted habitable range can be described. For *P. anubis*, from an initial restricted predicted habitable range, a gradual increase is seen through the Late Pleistocene to a maximum extent at the LGM (ca. 11 million km^2^), followed by a steep decline shortly afterwards. This contrasts with the range of *P. papio* where sharp peaks are observed at the start of the Late Pleistocene (ca. 5.5 million km^2^) and Holocene (ca. 4 million km^2^), with a very limited predicted habitable range through most of the glacial-interglacial cycle (average ca. 1.6 million km^2^). Both *P. kindae* and the *Papio* genus show a comparable pattern, with higher amplitude oscillations in the predicted habitable range during the first half of the Late Pleistocene, leading to a sustained maximum predicted habitable range prior to the LGM (*P. kindae* maximum ca. 3.3 million km^2^; the *Papio* genus maximum ca. 16.4 million km^2^), followed by a steadier decline than seen for *P. anubis*. *Both P. cynocephalus* (maximum ca. 8.5 million km^2^) and P. *hamadryas* (maximum ca. 1.4 million km^2^) show a clearer pattern of oscillation through the whole glacial-interglacial cycle, but with a sustained trough in predicted habitable range for *P cycnocephalus* leading up to the LGM, contrasting with a sustained peak for *P. hamadryas* with more limited troughs in predicted habitable ranges until the onset of the Holocene. In contrast to other species, the relatively limited scope of change for *P. ursinus* with isolated but pronounced peaks in the predicted habitable range is notable (maximum ca. 2.1 million km^2^). Importantly, these results illustrate that inferring baboon refugia based solely on comparisons between the present day and the LGM would typically fail to capture true peaks and troughs in the extent of predicted habitable ranges, with significant relationships between predicted habitable ranges with obliquity and precession indicating the importance of considering the influence of different tempos of orbitally driven climate change. This validates an approach that focuses on a full glacial-interglacial cycle to identify climatic refugia.

**Fig. 5:**
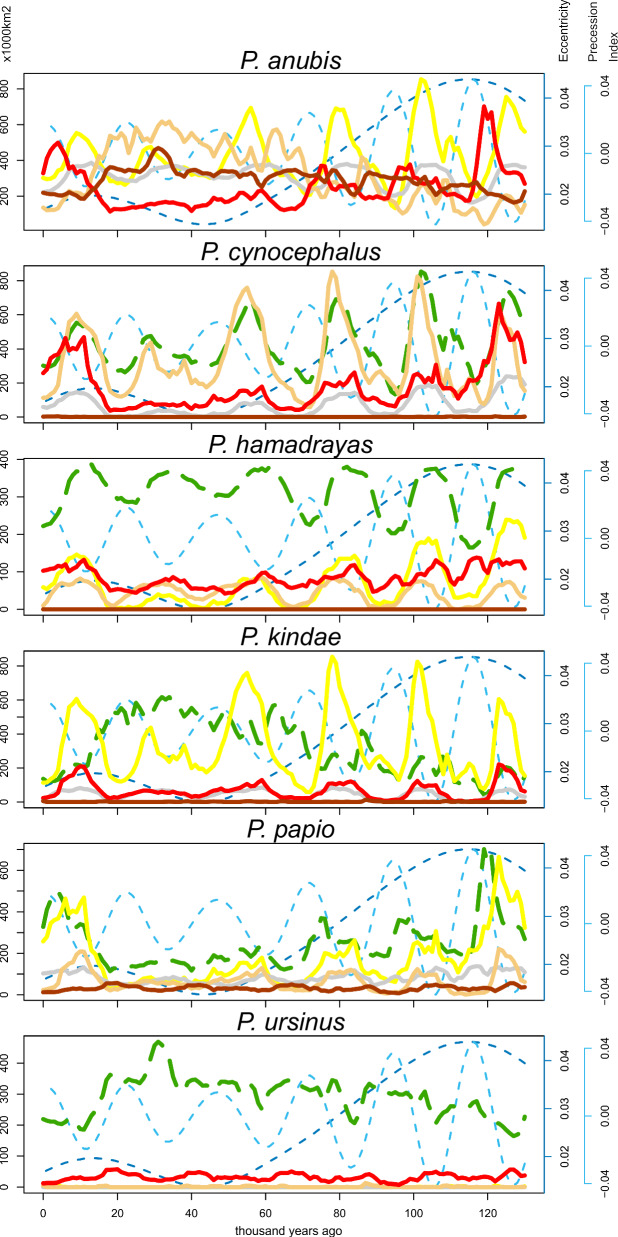
Graphs illustrating extent of overlap in predicted habitable range for each species through the Late Pleistocene and Holocene. Area (1000 km^2^) of overlap between the predicted habitable range of species through time, with respect to eccentricity (dashed dark blue) and precession (dashed light blue).

We examined predicted overlaps between species through time, based on the *Summed* predicted habitable range (Fig. 5). Overlaps between the *Summed* predicted habitable range of species are clearly influenced by the combinations of orbital parameters influencing the predicted habitable ranges of individual species, as well as patterns of geographic proximity, with the results of linear regressions between predicted overlaps in pairs of baboon species with eccentricity, obliquity, precession and insolation presented in Supplementary Data 2. Most widespread overlaps occur with *P. anubis*, related to the large extent of its predicted range, typically ranging between 250–450 thousand km^2^, with minimum overlaps ranging from 42,000 km^2^ (*P. kindae*) and 165,000 km^2^ (*P. hamadryas*). Notable peaks of overlap with *P. anubis* with *P. cynocephalus* (854,000 km^2^) and *P. papio* (702,000 km^2^) occur in Marine Isotope Stage (MIS) 5, and a phase of extended overlap with *P. kindae* in MIS 3 (up to 617,000 km^2^) is observed. Similar peaks are shared between *P. cynocephalus* with *P. kindae* (854,000 km^2^), and *P. papio* (665,000 km^2^), with more muted peak overlaps between *P. kindae* and *P. papio* occurring in early MIS 5 and MIS 1 (221,000 km^2^). *P. ursinus* only shares substantive overlaps with *P. anubis*, peaking ca. 30 ka, with more consistent limited overlaps with *P. papio*, and typically no overlaps observed with other species. The most extensive overlaps with *P. hamadryas* ranges occur with *P. anubis*, between 387 and 165 thousand km^2^ with multiple peaks throughout the Late Pleistocene and Holocene, with range overlaps with other species spanning 239,000 km^2^ for *P. cynocephalus*, in early MIS 5, to repeated periods of no overlaps with *P. kindae*, and no overlaps at all with *P. ursinus*.

## Discussion

Our ensemble models for the present distribution of baboons, both at a genus and individual species level, allow us to delimit their predicted habitable range throughout the Late Pleistocene and Holocene and enable us to identify and describe areas of persistent habitability as refugia. The results indicate widespread habitat suitability across sub-Saharan Africa at a genus level, with the presence of large, contiguous refugia zones spanning the eastern African Rift Valley system and along the south and south-western African coast, alongside isolated upland refugia in Arabia and western Africa, with a comparable pattern observed for *P. anubis*. Smaller predicted refugia for *P. hamadryas* and *P. ursinus* closely correspond to the present distribution of these species, whereas geographically stable refugia for *P. cynocephalus* and *P. papio*, are highly limited, and absent for *P. kindae*. This patterning at a 30-arc-minute resolution is broadly replicated by analyses conducted at 10-arc-minute resolution, indicating our results are robust to differences in spatial resolution. The present distribution of baboons, as well as the distribution of fossil baboon specimens, closely corresponds to the refugia predicted at a genus level, matching our evaluation of the scale of predicted habitable range through time with the 0 ka timeslice close to the minimum predicted habitable range for the Late Pleistocene and Holocene.

Several previous studies have used SDM approaches to explore the distribution of current habitat suitability for baboons^26,55^. Chala and colleagues^56^ extend this to include the projection of present habitat preferences to LGM climatic conditions, though direct comparisons with this study are limited by differences in data resolution and methods. At a genus level, we predict a wider presence of refugia in the Albertine Rift, disconnection between refugia in the eastern African Rift System and southern Africa, and small isolated refugia spanning western Africa. This contrasts with the shared suitability of habitats between the present and LGM presented by Chala and colleagues^56^

which indicates more extensive suitable habitats in southern central Africa and spanning western Africa. At a species level, Chala and colleagues^56^ predict more restricted ranges for all species except for *P. ursinus*, with relatively limited overlaps between species, contrasting with our prediction for extensive overlaps in the predicted habitable range, especially in tropical Africa. These differences may, in part, be accounted for by the difference in chronological resolution between comparing two time-slices (present and LGM) and diachronic change throughout a glacial-interglacial cycle, and the role that orbitally driven climate change may play in modulating the distribution of the predicted habitable range of baboon species.

Our results provide evidence^56^ for periods where the predicted habitable range of baboons extends around the Red Sea coastline. Such areas of habitability, which are not part of the long-term refugia inferred with the *Step-Wise* or *Summed* approach we have focused on in this paper, are directly related to modern and historic distributions. However, wider areas of predicted habitability, particularly for *P. ursinus* (Supplementary Movie 6) and to a lesser extent *P. hamadryas* (Supplementary Movie 4), do support potential habitability for baboons around the Red Sea coastline. The close genetic relationship between *P. hamadryas* populations in coastal Eritrea and the Arabian peninsula^46^ suggests the possible colonisation of the latter via the Bab-al-Mandeb. However, the presence of two discrete mitochondrial clades in Arabia also suggests the possibility of dispersal via both the northern (Sinai) and southern (Bab-al-Mandeb) routes^45,46^, which is supported by patterns of predicted habitable range for the genus.

The distribution of predicted refugia for baboons we identify shares similarities with those predicted for a range of other savannah taxa, which typically include a longitudinally-oriented refugium in the East African Rift Valley system, and a latitudinally-oriented refugium spanning West Africa^25^. Major suture zones between ungulate taxa are observed both between western and eastern African refugia, such as for African buffalo (*Syncerus caffer*), waterbuck (*Kobus ellipsiprymnus*), kob (*Kobus kob*), and bush pigs (*Potamochoerus* sp.) as well as between the northern and southern Rift Valley, such as for hartebeest (*Alcelaphus* sp.), common eland (*Taurotragus oryx*) and common warthog (*Phacocoerus africanus*), which share comparisons with the distribution of baboon species^25,57^. Baboons also share the presence of discrete southern/south-western refugia with a number of other savannah taxa. Our analysis and comparison to previous modelling work for baboons highlights that comparisons between the LGM and present-day may not fully capture peaks and troughs of potential distribution for the baboon species. Examination of a full glacial-interglacial cycle may be important to accurately identify refugia for a broader range of savannah taxa with geographically structured populations, such as giraffe^58^.

Recent studies have illuminated considerable complexity in baboon population genetics^32,59^, including geographically mediated relationships between species and gene flow among populations mainly via male dispersal. Broad differentiation of baboon species occurred within the Middle Pleistocene, beginning ca. 1.5 million, coincident with divergence amongst early *Homo* populations, and resolving the present configuration of species by ca. 150,000 years ago^59^. Our identification of discrete southern refugia for *P. ursinus* in southern Africa is consistent with the southern group (mtDNA-A) as an outgroup to other mt-lineages^30^, evidence for population continuity in the region over the Holocene^60^, and the limited overlaps in predicted distributions over the Late Pleistocene and Holocene (Fig. 5). Despite the absence of geographically stable refugia, repeated and extensive overlaps between the predicted habitable range for *P. cynocephalus* and *P. kindae* (Fig. 5) are consistent with the close genetic relationship between these species, evident in both mtDNA and Y-chromosome data^30^. The close genetic relationship between northern *P. ursinus* and southern *P. cynocephalus* may be explained by geographic proximity, introgression and nuclear swamping^29,30,33^, though both share the genus-level refugia identified in the southern extent of the East African Rift Valley^30^. A broadly east-west differentiation of *P. hamadryas*, discrete lineages of *P. anubis*, and *P. papio* can be observed in mtDNA studies^30^, corresponding to the distribution of predicted refugia as identified at both species and genus levels. The regular overlaps in the predicted habitable range between *P. anubis* and *P. hamadryas* we describe (Fig. 5) suggest introgressive hybridisation between these species may have been a recurrent feature throughout the Late Pleistocene and Holocene. Future studies can elaborate on the extent to which shared or sole occupation of the refugia we identify can better explain patterns of phylogeography than patterns of geographic proximity. Examination of hybrid zones suggests limited genetic barriers to crossbreeding between species, even spanning substantial differences in social organisation and mating system, such as between polygamous *P. anubis* and *P. hamadryas* or *P. papio*, where single male breeding units dominate^61^. The absence of clear genetic barriers to cross-breeding may, in part, relate to the relatively short time baboons have been constrained to and differentiated within savannah refugia alongside recent periods of extensive potential introgression between multiple parapatric species and populations.

The extensive predicted habitable range for *Papio* spanning much of sub-Saharan Africa at some point within the last interglacial-glacial cycle offers a potential analogue for early hominins where climatic tolerances are shared. The extensively predicted refugia in the Rift Valley for baboons may corroborate the important role the region has played for early hominin populations. In particular, the significant predicted refugia in western and south-western Africa are notable and have received less attention from palaeoanthropologists, despite emerging research increasingly pointing to the time depth of an early human presence here^62–64^. High amplitude changes in predicted habitable range overlaps between baboon species (Fig. 5), particularly between *P. anubis*, *P. cynocephalus, P. kindae* and *P. papio*, modulated by precessional cycles, offer potential insight into patterns of connectivity and isolation that may be particularly relevant for exploration of patterns of population structure for hominins^47,48^. The potential for terrestrial routes of dispersal for *Papio* around the Red Sea as opposed to a maritime crossing of the Bab al Mandeb strait discussed above share parallels with debates surrounding expansions of Late Pleistocene expansions from Africa to Arabia^65–69^. Whilst an initial terrestrial expansion from Africa to Arabia via a northern route with subsequent back-migration to Africa via a southern route has been proposed for both *P. hamadryas* and *H. sapiens*^43,56^, higher genetic diversity evident in southern Arabian *P. hamadryas* populations in contrast to northern populations is more consistent with the use of southern routes of expansion^43^.

Here, we have examined patterns of habitability throughout a full glacial-interglacial cycle (Late Pleistocene), and between the LGM and Holocene, which are typical timeframes for exploration of population refugia, rooted in the dominant role that eccentricity cycles have played in driving climatic variability. Our models suggest that all baboon species respond to patterns of eccentricity, whether this drives increases in predicted habitable ranges (best expressed by *P. anubis*, *P. ursinus*, and the *Papio* genus) or modulates the amplitude of changes to predicted habitable ranges. Precessional cycles clearly play an important role in driving peaks and troughs of predicted habitable ranges, particularly for the tropical species of *P. cynocephalus*, and *P. kindae*, as well as for *P. papio*, whereas obliquity appears to have an influence over the distribution patterns of eastern African species, *P. anubis*, *P. cynocephalus*, and *P. hamadryas*. While differences between the LGM and modern conditions sample some of the flux in environments experienced by *Papio*, and perhaps other savannah species, a longer-term view of a full interglacial-glacial cycle can better capture maxima and minima in predicted habitable ranges, and thus offer a more reliable means to identify refugia. Notably, examining patterns of flux with shorter periodicity may help illuminate the importance of identifying refugia. For example, *P. cynocephalus* typically occupies a restricted distribution, with shorter pulses of expansion, contrasting with *P. hamadryas* which typically occupies an expanded distribution with shorter pulses of contraction, implying refugia may play different roles between species. Finally, a longer-term view of the patterning of baboon distributions in relation to past climatic change may offer important context to understand how alternate climate futures may impact their range, given that today each baboon species occurs close to a predicted minimum in range distribution.

## Methods

### Taxonomy of baboons

The taxonomy of baboons has been debated for decades^70^. Genomic studies support at least six evolutionary units that followed largely independent evolutionary trajectories^32,59,71^. The taxonomic ranking of these units depends on the applied species concept. We regard baboons here as a group of six phylogenetic species.

### Data source

We compiled a dataset from both modern (since 1991; *n* = 777) and recent historic (1800–1990; *n* = 625) locations of baboon presence, recording the latitude, longitude, and species (Supplementary Data 1). Current occurrences predominantly (*n* = 710) represent primary recording of GPS locations in the field by members of the German Primate Centre, with the remaining occurrences recorded on field maps, from which geographic coordinates were subsequently derived. Historical occurrence data stem from museum specimens (*n* = 559) and the literature (*n* = 66). The corresponding geographic coordinates of most historical occurrences (*n* = 548) came directly from museum catalogues or secondary literature^72^. For the remaining occurrences (*n* = 77), the geographic origins were indicated on maps (*n* = 66)^73^ or by other geographic data (*n* = 11) from which we subsequently determined the geographic coordinates. Historical data were checked for plausibility of geographic origins, excluding instances with coordinates beyond land margins or in major cities.

### Species distribution modelling

Ensemble modelling was conducted using *tidysdm*^50^ in R. Our modern and historic presence dataset was thinned to ensure only a single presence per cell was employed in the modelling. We generated a pseudo-absence dataset six times the size of the presence dataset for each species, bounded by the limits of continental Africa and Arabia, and extending between 4 and 6 degrees beyond the maximum and minimum latitude and longitude coordinates of each species (See Supplementary Data 1). We employed a climate model and ancillary datasets from Krapp and colleagues^51^ at their native 30 arc-minute resolution (55.6 km square cells), as well as delta-downscaled to a resolution of 10 arc-minutes (18.5 km square cells), through *pastclim*^52^. Briefly, the climate reconstructions are based on a statistical emulator of the HadCM3 global circulation model, downscaled using the delta method. Predictor variables were selected with at least a 25% non-overlapping distribution between presence and pseudoabsences and with correlations lower than 0.7 to limit the impact of multicollinearities. Comparable model performance was achieved using a single suite of variables for all baboon species, based on those selected for the whole genus, and for the selection of predictor variables on a species-by-species basis; we employed model ensembles constructed with species-specific variables to offer insight into which factors may drive differences in predicted habitable range and distribution of potential refugia, whilst the shared suite of variables were employed at the genus level. We created ensemble models including generalised linear models (GLM), random forest, generalised boosted regression models (GBM), and maximum entropy (MaxEnt) methods, employing 4-fold spatial block cross-validation to choose the most appropriate model hyperparameters, as well as to assess the goodness of fit. Individual models were added to the ensemble based on the Boyce continuous index, with a minimum threshold of 0.7 for inclusion, and taking the median of available model predictions prior to employing a binary threshold, based upon the maximum sum of sensitivity and specificity (i.e., maximising TSS). The ensemble models were then used to predict binary habitable potential for each species throughout the Late Pleistocene and Holocene, with potentially habitable cells coded as 1. We note that SDM predictions represent the predicted habitable range of a species based on its climatic niche; however, certain parts of the predicted habitable range might not have been inhabited due to barriers to dispersal, or biotic interactions (e.g., competition with other species^74^). Comparable results were returned by analyses at 30 arc-minutes and 10 arc-minutes (See Supplementary Information; Supplementary Data 2), consistent with recent analysis indicating finer scales of spatial resolution do not inherently improve the utility of climate model datasets^75^.

### Predicted habitable range and refugia modelling

We employed two approaches to constrain the predicted habitable range of baboon species and identify the refugia they contain throughout the Late Pleistocene and Holocene, aiming to exclude areas that were predicted to be potentially habitable but without clear connectivity to the present-day range of baboons. Our step-wise approach selected and retained contiguous patches of cells that were predicted to be potentially habitable (i.e., with a value of 1) in the 0 ka timestep that overlapped with presences in the observed dataset. Contiguous patches of potentially habitable cells in the 1 ka timestep were selected and retained where they shared at least partial overlap with those cells retained for the 0 ka timestep. This process was iterated in a step-wise fashion from 1 ka to 130 ka, yielding 131 time-slices with retained potentially habitable cells coded as 1. Our summed approach added all 131 binary time-slices into a single raster, where cell values represented the count of time-slices that were predicted to be potentially habitable. Contiguous patches of summed potentially habitable cells were then selected and retained where they overlapped with presences in the observed dataset. In each case, predicted refugia were identified where cells were predicted to be part of the habitable range in all 131 time slices examined. Each time slice can be viewed as part of the GIFs for each species as Supplementary Movies.

### Reporting summary

Further information on research design is available in the Nature Portfolio Reporting Summary linked to this article.

## Supplementary information

Supplementary Information

Description of Additional Supplementary Files

Supplementary Data 1

Supplementary Data 2

Supplementary Movie 1: *Papio* model through time

Supplementary Movie 2: *P. anubis* model through time

Supplementary Movie 3: *P. cynocephalus* model through time

Supplementary Movie 4: *P. hamadryas* model through time

Supplementary Movie 5: *P. kindae* model through time

Supplementary Movie 6: *P. papio* model through time

Supplementary Movie 7: *P. ursinus* model through time

Reporting Summary

## Data Availability

Raw data are supplied as Supplementary Data 1.

## Supplementary information

ODMAP Protocol Document and comparison of results of SDM and refugia analysis at 30 arc-minute and 10 arc-minute resolutions. The online version contains supplementary material available at 10.1038/s42003-025-08419-8.

## Code availability

Analytical code is accessible at https://github.com/jblinkhorn/Papio-Refugia.
