# Supplementary material for: Identifying late Pleistocene and Holocene refugia for baboons
Source: Commun Biol. 2025 Jul 4;8:1003. doi: 10.1038/s42003-025-08419-8 (PMC12227712; doi:10.1038/s42003-025-08419-8)
Supplement: Supplementary file 1 — Supplementary Information [file 42003_2025_8419_MOESM1_ESM.pdf]

**Identifying Late Pleistocene and Holocene refugia for baboons**

James Blinkhorn[1,2], Dietmar Zinner[3,4,5], Lucy Timbrell[1,2], Andrea Manica[6], Matt Grove[1], Eleanor M. L. Scerri[2,7,8]

[1]Department of Archaeology, Classics, and Egyptology, University of Liverpool, Liverpool, U.K.

[2]Human Palaeosystems Group, Max Planck Institute of Geoanthropology, Jena, Germany

[3]Cognitive Ethology Laboratory, German Primate Center, Leibniz Institute for Primate Research, Göttingen, Germany

[4]Department of Primate Cognition, Georg-August-Universität Göttingen, Göttingen, Germany

[5]Leibniz ScienceCampus Primate Cognition, Göttingen, Germany

[6]Department of Zoology, University of Cambridge, Cambridge, United Kingdom

[7]Department of Classics and Archaeology, University of Malta, Faculty of Arts; Msida, Malta

[8]Institute of Prehistoric Archaeology, University of Cologne; Cologne, Germany

Corresponding Authors: J. Blinkhorn (j.blinkhorn@liverpool.ac.uk; blinkhorn@gea.mpg.de); E. Scerri (scerri@gea.mpg.de)

James Blinkhorn Orcid ID: 0000-0002-9399-5515

Dietmar Zinner Orcid ID: 0000-0003-3967-8014

Lucy Timbrell Orcid ID: 0000-0003-1229-554X

Andrea Manica Orcid ID: 0000-0003-1895-450X

Matt Grove Orcid ID: 0000-0002-2293-8732

Eleanor Scerri Orcid ID: 0000-0002-7468-9977

**Supplementary Information:**

## ODMAP Statement

### Overview

**Authorship**

Title: Identifying Late Pleistocene and Holocene refugia for baboons

Authors: James Blinkhorn, Dietmar Zinner, Lucy Timbrell, Andrea Manica, Matt Grove, Eleanor Scerri

Contact: j.blinkhorn@liverpool.ac.uk; scerri@gea.mpg.de

Study Link:

**Model Objective**

Model Objective: Transfer

Target Output: Maps of binary probability of habitat suitability

**Taxon**

Focal Taxon: Baboons; (*Papio)*

**Location**

Location: Continental Africa and Arabia

**Scale of Analysis**

Spatial Extent: -17.5, 60, -35, 37.5 (xmin, xmax, ymin, ymax)

Spatial Resolution: 0.5, 0.167

Temporal Extent: 0 – 130000 years ago

Temporal Resolution: 1000 years

Boundary: Political

**Biodiversity Data Overview**

Observation type: Field survey; historical field survey

Response data type: presence data only

Types of Predictors: bioclimatic, topographic

**Conceptual Model**

Hypotheses: We employ bioclimatic and topographic variables in the SDM's as we anticipate they can characterise the habitable range for baboon populations.

**Assumptions**

Assumptions:

1. Species–environment equilibrium: Baboon distribution is driven in part by (or correlated with) bioclimatic and topographic variability.
2. Observation Bias: We assume sampling is adequate and representative
3. Independence of species observations: Each observation record represents discrete information
4. Availability of all important predictors: Key explanatory bioclimatic and topographic variables available for modelling; additional ecological variables (NPP; LAI) that are derived from bioclimatic modelling were excluded in final modelling.
5. Predictors are free of error:
6. Niche stability: We assume that species retain their niche across space and time
7. Extrapolation issues: We assume that the relationship between species observation and predictor variables apply when transfer to the past, without significant change to the correlations between environmental variables.

**SDM algorithms**

Model algorithms: GLM, Random Forest (RF), Boosted Trees (GBM), MaxEnt

Justification of model complexity: Preliminary analyses using individual model algorithms achieved high model accuracy (based on AUC and TSS), but while substantive model overlaps were achieved, some methods predicted more expansive patterns of habitability than others.

Is ensemble modelling used?: Yes

**Model Workflow**

**Software, codes, and data**

Software: R v.4.4.1, tidysdm v.0.9.5[1] , pastclim v. 2.1.0.9002[2]

Code: Analytical Code is accessible at: https://github.com/jblinkhorn/Papio-Refugia

Data: Raw presence data are provided as Supplementary Information 1

## Data

**Biodiversity Data**

Taxon names: *Papio, Papio anubis, Papio cynocephalus, Papio hamadryas, Papio kindae, Papio papio, Papio ursinus*

Taxonomic reference system:

Ecological Level: genus, species

Biodiversity data sources and sampling design: Primary data were collected by members of the German Primate Centre. Secondary data were collected from museum sources, with a low proportion of data derived from the literature. Historical data were checked for plausibility of geographic origins, excluding instances with coordinates beyond land margins or in major cities.

Sample size per taxon: *P. anubis*: n=500; *P. cynocephalus*: n=146; *P. hamadryas*: n=194; *P. kindae*: n=113; *P. papio*: n=156; *P. ursinus*: 292; all *Papio*: n=1401

30 arc-minute Unique Cell Sample size per taxon: *P. anubis*: n=300; *P. cynocephalus*: n=77; *P. hamadryas*: n=89; *P. kindae*: n=81; *P. papio*: n=50; *P. ursinus*: 179; all *Papio*: n=758

10 arc-minute Unique Cell Sample size per taxon: *P. anubis*: n=407; *P. cynocephalus*: n=107; *P. hamadryas*: n=150; *P. kindae*: n=100; *P. papio*: n=80; *P. ursinus*: 245; all *Papio*: n=1081

Country/region mask: NA

Details on scaling: NA

Data cleaning/filtering: Presence data were thinned to include only a single presence per raster cell

Absence data collection: None

Pseudo-absence and background data: Background points were generated within an area equal to the maximum bounding rectangle around presence points then expanded by 6 degrees in each direction for the analysis at 30-arc second resolution; for analysis at 10 arc-second resolution the maximum bounding rectangle was expanded by 6 degree except for *P. kindae* (5 degrees) and *P. papio* (4 degrees) due to calculation errors for the Generalised Linear Models. Six times as many background points for presence points for each analyses selected to optimise model performance statistics.

Potential errors and biases in data:

**Data Partitioning**

Selection of training and test data: We employed a spatial block cross validation scheme, splitting presence and background data into 4 folds for training, model tuning, and assessment, evaluating 10 combinations of hyperparameters per model where required.

**Predictor Variables**

Predictor variables used: Bio01, Bio04, Bio05, Bio06, Bio07, Bio08, Bio09, Bio10, Bio11, Bio12, Bio13, Bio14, Bio15, Bio16, Bio17, Bio18, Bio19, rugosity

Data sources: Krapp et al. 2021[3]; pastclim[2]

Spatial resolution and extent of raw data: 0.5; -180, 180, -180, 180 (xmin, xmax, ymin, ymax)

Map projection: EPSG:4326

Temporal resolution and extent of raw data: 1000 time slices, 0-799,000

Data processing and scaling: To evaluate whether an increase in spatial resolution would significantly impact the outcome of the analyses, we applied a delta-downscaling method to increase spatial resolution from 30 to 10 arc minutes using tools available in the pastclim package.

Measurement errors and bias: NA

Dimension reduction: No dimension reduction methods employed

**Transfer Data for projection**

Data sources: Krapp et al. 2021;

Spatial resolution and extent: 0.5; -180, 180, -180, 180 (xmin, xmax, ymin, ymax)

Temporal resolution and extent: 1000-year time slices, 0-799,000 years

Models and scenarios used: Palaeoclimate reconstructions based on the HadCM3 model, bias corrected for the CRU observations dataset used for the modelling, see Krapp et al. 2021[3];

Data processing and scaling: To evaluate whether an increase in spatial resolution would significantly impact the outcome of the analyses, we applied a delta-downscaling method to increase spatial resolution from 30 to 10 arc minutes using tools available in the pastclim package.

Quantification of novel environments: NA

## Model

**Variable Selection:** Predictor variables with a dissimilarity of >0.2 between presence and background points were selected for assessment of multicollinearity

**Multicollinearity:** We used the filter-collinear tool in tidysdm to remove selected variables that exhibit high correlations, using a filter cutoff of 0.7

**Model settings/model complexity:**

| Metric | Spatial Resolution | *P. anubis* | *P. cynocephalus* | *P. hamadryas* | *P. kindae* | *P. papio* | *P. ursinus* | *Papio* |
|---|---|---|---|---|---|---|---|---|
| Mean Boyce Continuous Index | 30 arc-minute | 0.878 | 0.768 | 0.796 | 0.752 | 0.624 | 0.742 | 0.922 |
| | 10 arc-minute | 0.866 | 0.796 | 0.876 | 0.611 | 0.781 | 0.736 | 0.913 |
| Mean AUC | 30 arc-minute | 0.817 | 0.802 | 0.935 | 0.826 | 0.853 | 0.816 | 0.823 |
| | 10 arc-minute | 0.787 | 0.829 | 0.933 | 0.785 | 0.853 | 0.833 | 0.833 |
| Mean TSS | 30 arc-minute | 0.510 | 0.544 | 0.787 | 0.600 | 0.689 | 0.546 | 0.510 |
| | 10 arc-minute | 0.468 | 0.581 | 0.768 | 0.549 | 0.633 | 0.576 | 0.517 |

| Resolution | 30 arc-minutes | | | | | | | 10 arc-minutes | | | | | | |
|---|---|---|---|---|---|---|---|---|---|---|---|---|---|---|
| Species | *P. anubis* | *P. cynocephalus* | *P. hamadryas* | *P. kindae* | *P. papio* | *P. ursinus* | *Papio* | *P. anubis* | *P. cynocephalus* | *P. hamadryas* | *P. kindae* | *P. papio* | *P. ursinus* | *Papio* |
| Annual Mean Temperature (Bio01) | 0.077 | - | - | - | - | - | 0.075 | 0.101 | - | 0.019 | - | - | 0.124 | 0.098 |
| Temperature Seasonality (Bio04) | - | - | 0.048 | 0.188 | - | - | - | - | 0.057 | 0.033 | - | - | - | - |
| Max Temperature of Warmest Month (Bio05) | - | - | - | 0.073 | - | - | - | - | - | - | 0.146 | - | - | - |
| Temperature Annual Range (Bio07) | - | 0.080 | - | - | - | - | - | - | - | - | - | - | - | - |
| Mean Temperature of Wettest Quarter (Bio08) | - | - | 0.032 | - | - | - | 0.066 | - | - | - | - | 0.221 | - | 0.077 |
| Mean Temperature of the Driest Quarter (Bio09) | - | 0.116 | 0.032 | - | - | 0.093 | 0.069 | - | 0.113 | 0.017 | - | 0.115 | - | - |
| Mean Temperature of Coldest Quarter (Bio11) | - | - | - | - | 0.101 | - | - | - | - | - | - | - | - | - |
| Mean Annual Precipitation (Bio12) | - | - | - | - | - | 0.234 | - | - | - | - | - | - | - | - |
| Precipitation of Wettest Month (Bio13) | 0.176 | 0.075 | - | - | - | - | 0.224 | 0.135 | 0.082 | - | - | - | - | 0.235 |
| Precipitation of Driest Month (Bio14) | 0.057 | - | 0.038 | - | 0.126 | - | 0.050 | 0.055 | - | 0.031 | - | 0.076 | - | 0.057 |
| Precipitation Seasonality (Coefficient of Variation)(Bio15) | 0.078 | 0.063 | - | - | 0.121 | - | 0.077 | - | - | 0.016 | 0.155 | - | - | - |
| Precipitation of Wettest Quarter (Bio16) | - | - | - | 0.131 | - | - | - | - | - | - | 0.201 | - | 0.316 | - |
| Precipitation of Driest Quarter (Bio17) | - | - | - | - | - | 0.084 | - | - | - | - | - | - | - | - |
| Precipitation of Warmest Quarter (Bio18) | - | 0.077 | - | 0.084 | - | - | - | - | 0.080 | - | - | - | - | - |
| Precipitation of Coldest Quarter (Bio19) | 0.091 | 0.127 | 0.073 | 0.199 | 0.042 | - | 0.053 | 0.120 | 0.153 | 0.030 | 0.082 | 0.043 | - | 0.061 |
| Rugosity | 0.113 | 0.067 | 0.207 | - | 0.093 | 0.219 | 0.202 | 0.137 | 0.073 | 0.239 | - | 0.104 | 0.198 | 0.246 |

**Model selection:**

Model selection strategy: Models are added to the ensemble based on the Boyce Continuous Index

Method for model averaging: Simple mean of the prediction probability from the best performing version of each algorithm (maximising the Boyce Continuous Index).

Ensemble method: Models are added to the ensemble based on the Boyce Continuous Index

Non-independence correction: Data were thinned to unique cells both for the 30 arc-minute and 10 arc-minute resolutions. Furthermore, spatial non-independence was addressed by spatial block cross-validation with 5 blocks, randomly assigned to 4 folds.

**Threshold Selection:** We employ a metric threshold optimised against maximum TSS values to produce binary predictions.

## Assessment

**Performance statistics:** Boyce Continuous Index, AUC, TSS

**Plausibility Check:** Models were rejected if extensive and persistent habitability were predicted for regions in which no evidence for baboon occupation is known (e.g. central Sahara).

## Prediction

### Prediction output

Prediction Unit: Binary estimation of potential habitability, based on TSS maximisation.

Post-processing steps: Individual binary predictions of potential habitability are combined in either a stepwise or summed fashion to only include contiguous areas of potential habitability either by timeslice (step-wise) or through the whole time series (summed) that overlap with the modern distribution of the studied taxon, excluding areas of potential habitability in which there is no link to the observed presence of the taxon.

**Uncertainty quantification:** NA

**Comparison of SDM and Refugia analysis between 30 arc-minute and 10 arc-minute resolutions**

In order to explore the impact of spatial resolution on modelling outcomes, we downscaled climate model datasets presented by Krapp and colleagues[3] from 30 arc-minutes (ca. 55.6km x 55.6km grid squares at the equator) to 10 arc-minutes (ca. 18.5km x 18.5km grid squares at the equator) using functions available in *pastclim*[2]. The full code can be accessed at https://github.com/jblinkhorn/Papio-Refugia, alongside key outputs including the downscaled dataset for ready evaluation. The Methods statement provided in the Main Text describes both sets of analysis, with the ODMAP statement presented as Supplementary Information 2 reporting analysis at both spatial scales.

Table S1 presents the key evaluation metrics for models at both spatial scales. Broadly comparable results are observed across the key metrics presented, with minor differences in results varying by species as to whether 30 arc-minute or 10 arc-minute resolutions are indicated to perform better.

**Table S1:** Comparison of key evaluation metrics for models at 30 arc-minute and 10 arc-minute resolutions.

| Metric | Spatial Resolution | *P. anubis* | *P. cynocephalus* | *P. hamadryas* | *P. kindae* | *P. papio* | *P. ursinus* | *Papio* |
|---|---|---|---|---|---|---|---|---|
| Mean Boyce Continuous Index | 30 arc-minute | 0.878 | 0.768 | 0.796 | 0.752 | 0.624 | 0.742 | 0.922 |
| | 10 arc-minute | 0.866 | 0.796 | 0.876 | 0.611 | 0.781 | 0.736 | 0.913 |
| Mean AUC | 30 arc-minute | 0.817 | 0.802 | 0.935 | 0.826 | 0.853 | 0.816 | 0.823 |
| | 10 arc-minute | 0.787 | 0.829 | 0.933 | 0.785 | 0.853 | 0.833 | 0.833 |
| Mean TSS | 30 arc-minute | 0.510 | 0.544 | 0.787 | 0.600 | 0.689 | 0.546 | 0.510 |
| | 10 arc-minute | 0.468 | 0.581 | 0.768 | 0.549 | 0.633 | 0.576 | 0.517 |

Table S2 identifies variable importance for each species model between the two spatial scales. Although some variability occurs in variable selection and importance, and number of key similarities exist, including the importance of rugosity and precipitation of the coldest quarter (Bio19) across the majority of species, a broader contribution from variables relating to precipitation seasonality than temperature seasonality.

Table S2: Comparison of variable importance for models at 30 arc-minute and 10 arc-minute resolutions.

| Resolution | 30 arc-minutes | | | | | | | 10 arc-minutes | | | | | | |
|---|---|---|---|---|---|---|---|---|---|---|---|---|---|---|
| Species | *P. anubis* | *P. cynocephalus* | *P. hamadryas* | *P. kindae* | *P. papio* | *P. ursinus* | *Papio* | *P. anubis* | *P. cynocephalus* | *P. hamadryas* | *P. kindae* | *P. papio* | *P. ursinus* | *Papio* |
| Annual Mean Temperature (Bio01) | 0.077 | - | - | - | - | - | 0.075 | 0.101 | - | 0.019 | - | - | 0.124 | 0.098 |
| Temperature Seasonality (Bio04) | - | - | 0.048 | 0.188 | - | - | - | - | 0.057 | 0.033 | - | - | - | - |
| Max Temperature of Warmest Month (Bio05) | - | - | - | 0.073 | - | - | - | - | - | - | 0.146 | - | - | - |
| Temperature Annual Range (Bio07) | - | 0.080 | - | - | - | - | - | - | - | - | - | - | - | - |
| Mean Temperature of Wettest Quarter (Bio08) | - | - | 0.032 | - | - | - | 0.066 | - | - | - | - | 0.221 | - | 0.077 |
| Mean Temperature of the Driest Quarter (Bio09) | - | 0.116 | 0.032 | - | - | 0.093 | 0.069 | - | 0.113 | 0.017 | - | 0.115 | - | - |
| Mean Temperature of Coldest Quarter (Bio11) | - | - | - | - | 0.101 | - | - | - | - | - | - | - | - | - |
| Mean Annual Precipitation (Bio12) | - | - | - | - | - | 0.234 | - | - | - | - | - | - | - | - |
| Precipitation of Wettest Month (Bio13) | 0.176 | 0.075 | - | - | - | - | 0.224 | 0.135 | 0.082 | - | - | - | - | 0.235 |
| Precipitation of Driest Month (Bio14) | 0.057 | - | 0.038 | - | 0.126 | - | 0.050 | 0.055 | - | 0.031 | - | 0.076 | - | 0.057 |
| Precipitation Seasonality (Coefficient of Variation)(Bio15) | 0.078 | 0.063 | - | - | 0.121 | - | 0.077 | - | - | 0.016 | 0.155 | - | - | - |
| Precipitation of Wettest Quarter (Bio16) | - | - | - | 0.131 | - | - | - | - | - | - | 0.201 | - | 0.316 | - |
| Precipitation of Driest Quarter (Bio17) | - | - | - | - | - | 0.084 | - | - | - | - | - | - | - | - |
| Precipitation of Warmest Quarter (Bio18) | - | 0.077 | - | 0.084 | - | - | - | - | 0.080 | - | - | - | - | - |
| Precipitation of Coldest Quarter (Bio19) | 0.091 | 0.127 | 0.073 | 0.199 | 0.042 | - | 0.053 | 0.120 | 0.153 | 0.030 | 0.082 | 0.043 | - | 0.061 |
| Rugosity | 0.113 | 0.067 | 0.207 | - | 0.093 | 0.219 | 0.202 | 0.137 | 0.073 | 0.239 | - | 0.104 | 0.198 | 0.246 |

Table S3 shows the extent of potential refugia identified, the maximum extent of the predicted habitable range during the Late Pleistocene and Holocene, and the size of the potential refugia in proportion to this maximum predicted habitable range. Many of the broad patterns observed at the coarser, 30 arc-minute resolution are preserved in the results of the finer, 10 arc-minute resolution analysis both in terms of the overall extent of predicted habitable ranges and patterning of its relationship to proposed refugia. The largest difference is seen for modelling of *P. papio*, which suggests much more extensive predicted habitable range at a 10 arc-minute resolution, discussed further below. The predicted habitable ranges for *P. cynocephalus* are in broad agreement between resolutions under the *Summed* approach, but differ substantially with the *Step-Wise* approach.

**Table S3:** Extent of potential refugia identified, the maximum extent of the predicted habitable range during the Late Pleistocene and Holocene, and the size of the potential refugia in proportion to this maximum predicted habitable range modelled at 30 and 10 arc-minute resolutions.

| Approach | Variable | Resolution (arc-minutes) | *P. anubis* | *P. cynocephalus* | *P. hamadryas* | *P. kindae* | *P. papio* | *P. ursinus* | *Papio* |
|---|---|---|---|---|---|---|---|---|---|
| *Summed* | Potential Refugia (1000 km2) | 30 | 2926 | 65 | 444 | 0 | 31 | 591 | 5326 |
| | | 10 | 3167 | 34 | 576 | 0 | 4865 | 609 | 4985 |
| | Predicted Habitable Range (1000 km2) | 30 | 16541 | 13541 | 1848 | 8696 | 9596 | 3345 | 19649 |
| | | 10 | 17060 | 12310 | 3267 | 6877 | 29017 | 2886 | 17876 |
| | Proportional Refugia % | 30 | 17.7 | 0.5 | 24 | 0 | 0.3 | 17.7 | 27.1 |
| | | 10 | 18.6 | 0.3 | 17.6 | 0 | 16.8 | 21.1 | 27.9 |
| *Step-Wise* | Potential Refugia (1000 km2) | 30 | 2255 | 6 | 367 | - | 9 | 591 | 5212 |
| | | 10 | 1719 | 4 | 328 | 0 | 71 | 606 | 4826 |
| | Predicted Habitable Range (1000 km2) | 30 | 16073 | 13153 | 1848 | - | 8514 | 3250 | 19267 |
| | | 10 | 16719 | 4188 | 3118 | 5856 | 28533 | 2782 | 17410 |
| | Proportional Refugia % | 30 | 14 | <0.1 | 19.8 | - | 0.1 | 18.2 | 27.1 |
| | | 10 | 10.3 | <0.1 | 10.5 | 0 | <0.1 | 21.8 | 27.7 |

In Figure S1, we compare the predicted habitable range and potential refugia for all *Papio*. The broad configuration of predicted refugia shows considerable similarity, with key differences relating to the extent of refugia predicted across both methods in the southern East African Rift and the frequency with which the predicted habitable range spans north-central and western Africa. Both results are consistent with the limited distribution of fossil baboons illustrated in Figure 1. At a species level (illustrated in Figure S2) a similar pattern is observed for the predicted habitable ranges across all populations. Similar configurations of potential refugia are seen for *P. anubis, P. cynocephalus*, *P. kindae*, and *P. ursinus*. A significantly extended predicted habitable range is observed for *P. hamadryas*, resulting in extensive potential refugia identified using the

Summed approach at 10 arc-minute resolution throughout the East African Rift Valley. Much more extensive predicted habitable range is observed for *P. papio*, resulting in patches of potential refugia that overlap with and extend beyond the distribution refugia predicted at a genus level.

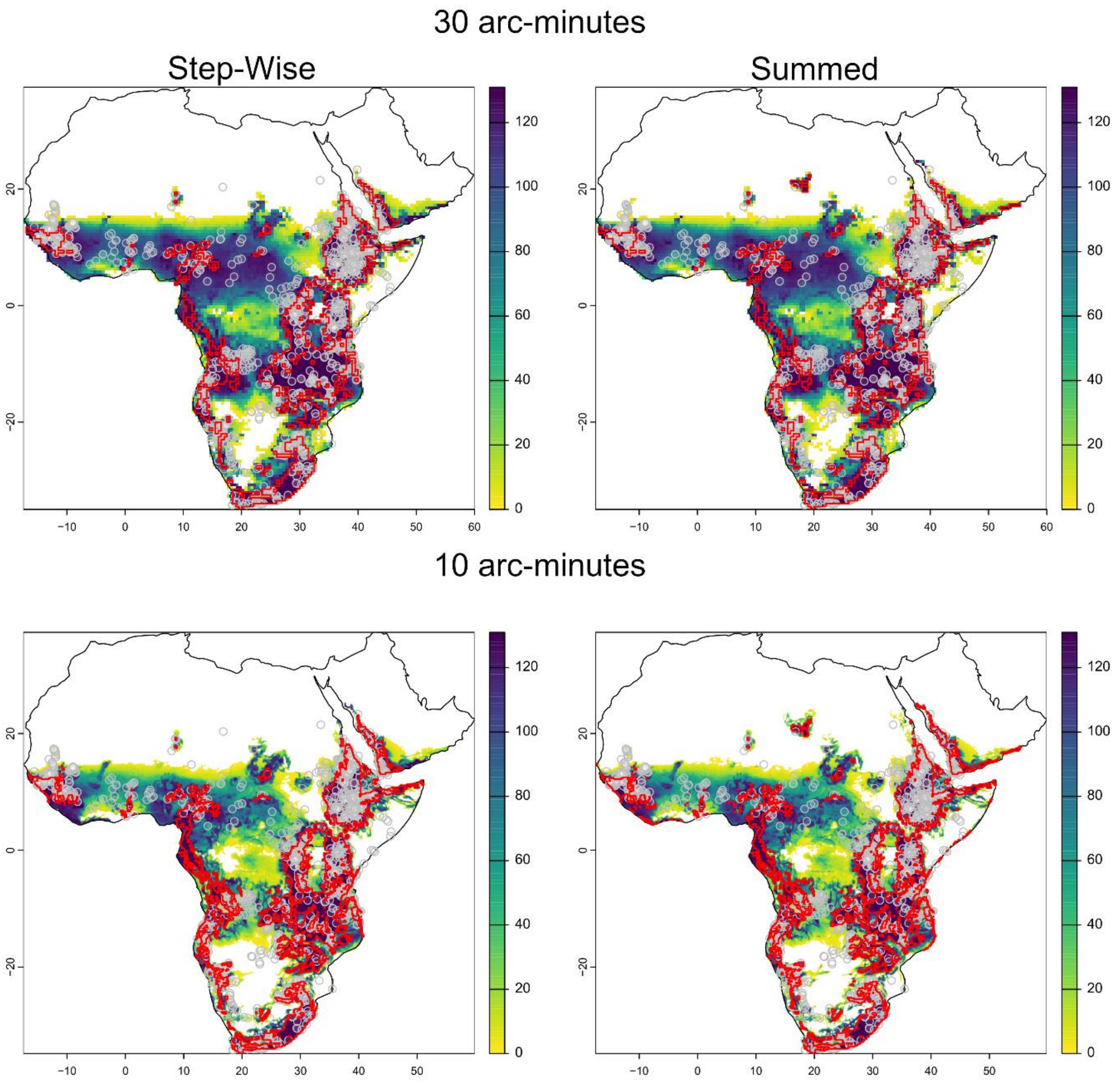


**Figure S1:** Predicted *Step-Wise* (left) and *Summed* (right) habitable range for the genus *Papio* at 30 arc-minute (top) and 10 arc-minute (bottom) resolution, with the colour scale illustrating the number of time-slices cells that are predicted to form part of the habitable range, with potential refugia (where cell count = 131) outlined in red; modern and recent historical presence data are illustrated as grey circles.

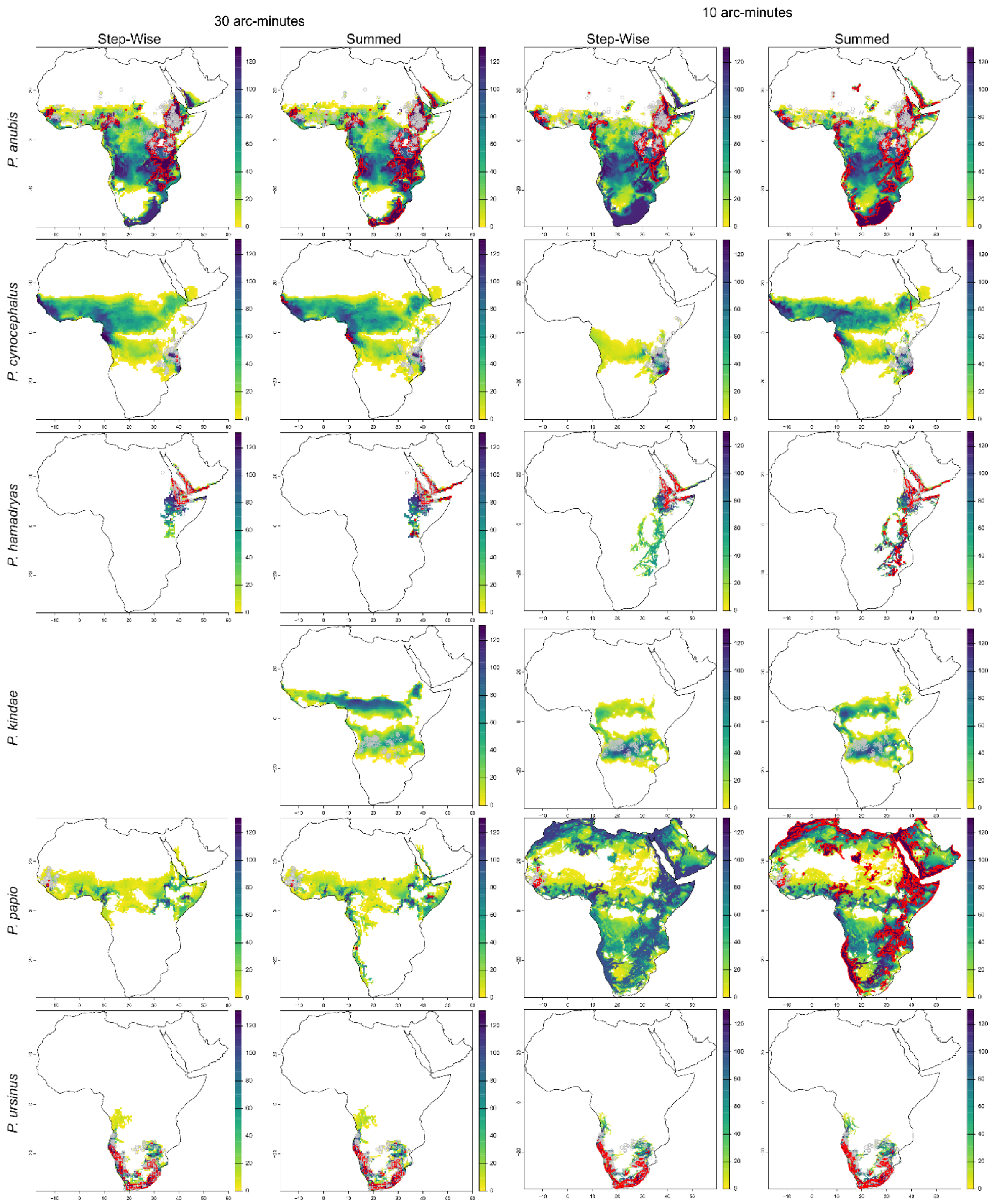


**Figure S2:** Predicted Step-Wise and Summed habitable range for each baboon species at 30 arc-minute and 10 arc-minutes, with the colour scale illustrating the count of time-slices cells that are predicted to form part of the habitable range, with potential refugia (where cell count = 131) outlined in red; modern and recent historical presence data are illustrated as grey circles. No Step-Wise continuity in predicted habitable range at 30 arc-minute resolution was identified for P. kindae.

Finally, in Table S4, we compare the results of regression analysis regression at alternate spatial resolutions between the extent of predicted habitable range of each baboon species through time and orbital parameters that influence climate change. Broad comparability is observed in the

results between alternate resolutions, with only five of twenty-eight comparisons returning differing results, three of which relate to differences in models for *P. papio*, indicating obliquity, rather than precssion and eccentricity sharing a significant relationship to the extent of predicted habitable range through time at 10 arc-minute resolution. Insolation shares a significant relationship to the predicted habitable range for *P. ursinus* at 10 arc-minute resolution, but this relationship is no longer significant for *P. hamadryas* at this resolution.

**Table S4:** Comparison of regression results between the extent of predicted habitable range of each baboon species through time and orbital parameters that influence climate change between 30 and 10 arc-minute resolutions.

| Species | Variable | Eccentricity | | Climatic Precession | | Obliquity | | Insolation | |
|---|---|---|---|---|---|---|---|---|---|
| | Resolution | 30 | 10 | 30 | 10 | 30 | 10 | 30 | 10 |
| *P. anubis* | r_squared | 0.29 | 0.31 | 0.00 | 0.00 | 0.14 | 0.15 | 0.00 | 0.01 |
| | f_statistic | 53.49 | 59.22 | 0.32 | 0.00 | 21.22 | 23.24 | 0.05 | 1.53 |
| | p_value | 0.00 | 0.00 | 0.57 | 0.97 | 0.00 | 0.00 | 0.83 | 0.22 |
| *P. cynocephalus* | r_squared | 0.18 | 0.28 | 0.42 | 0.45 | 0.11 | 0.11 | 0.01 | 0.01 |
| | f_statistic | 28.49 | 51.28 | 92.23 | 103.85 | 15.83 | 16.65 | 0.62 | 1.66 |
| | p_value | 0.00 | 0.00 | 0.00 | 0.00 | 0.00 | 0.00 | 0.43 | 0.20 |
| *P. hamadryas* | r_squared | 0.22 | 0.34 | 0.59 | 0.04 | 0.05 | 0.09 | 0.14 | 0.00 |
| | f_statistic | 35.85 | 66.71 | 188.10 | 4.68 | 6.16 | 13.26 | 20.25 | 0.62 |
| | p_value | 0.00 | 0.00 | 0.00 | 0.03 | 0.01 | 0.00 | 0.00 | 0.43 |
| *P. kindae* | r_squared | 0.60 | 0.39 | 0.05 | 0.14 | 0.00 | 0.00 | 0.00 | 0.02 |
| | f_statistic | 191.47 | 82.53 | 6.77 | 21.15 | 0.37 | 0.01 | 0.41 | 3.09 |
| | p_value | 0.00 | 0.00 | 0.01 | 0.00 | 0.54 | 0.94 | 0.53 | 0.08 |
| *P. papio* | r_squared | 0.28 | 0.02 | 0.07 | 0.01 | 0.00 | 0.05 | 0.13 | 0.06 |
| | f_statistic | 49.56 | 3.08 | 10.30 | 1.38 | 0.16 | 7.3 | 19.94 | 8.74 |
| | p_value | 0.00 | 0.08 | 0.00 | 0.24 | 0.69 | 0.01 | 0.00 | 0.00 |
| *P. ursinus* | r_squared | 0.34 | 0.14 | 0.06 | 0.19 | 0.01 | 0.00 | 0.01 | 0.07 |
| | f_statistic | 66.59 | 21.06 | 8.01 | 29.44 | 1.80 | 0.22 | 1.09 | 10.14 |
| | p_value | 0.00 | 0.00 | 0.01 | 0.00 | 0.18 | 0.64 | 0.30 | 0.00 |
| *Papio* | r_squared | 0.34 | 0.07 | 0.06 | 0.44 | 0.01 | 0.01 | 0.01 | 0.04 |
| | f_statistic | 66.59 | 9.48 | 8.01 | 103.22 | 1.80 | 1.23 | 1.09 | 4.87 |
| | p_value | 0.00 | 0.00 | 0.01 | 0.00 | 0.18 | 0.27 | 0.30 | 0.03 |

Overall, we identify significant comparability in the analyses undertaken at 30 arc-minute and 10 arc-minute scales. Given the significant temporal and spatial scale of the analysis we present, we consider the analysis at 30 arc-minute resolution to offer a more conservative appraisal of potential baboon refugia. Nevertheless, examining the differences between spatial and temporal scales in models of predicted habitable range in further detail in the future will likely offer a profitable means to enhance appraisals of potential refugia.

**References Cited:**


1. Leonardi, M., Colucci, M., Pozzi, A. & Manica, A. tidysdm: Species Distribution Models with Tidymodels. R package version 0.9.5. (2024).
2. Leonardi, M., Hallett, E. Y., Beyer, R., Krapp, M. & Manica, A. pastclim 1.2: an R package to easily access and use paleoclimatic reconstructions. *Ecography (Cop.).* **2023**, e06481 (2023).
3. Krapp, M., Beyer, R. M., Edmundson, S. L., Valdes, P. J. & Manica, A. A statistics-based reconstruction of high-resolution global terrestrial climate for the last 800,000 years. *Scientific Data* **8**, 228 (2021).
