# Supplementary material for: Identifying late Pleistocene and Holocene refugia for baboons
Source: Commun Biol. 2025 Jul 4;8:1003. doi: 10.1038/s42003-025-08419-8 (PMC12227712; doi:10.1038/s42003-025-08419-8)
Supplement: Supplementary file 2 — Description of Additional Supplementary Files [file 42003_2025_8419_MOESM2_ESM.pdf]

Description of Additional Supplementary Files

File name: Supplementary Data 1

Description: A spreadsheet of modern and historical presence points of baboon species is presented as Supplementary Information.

File name: Supplementary Data 2

Description: A spreadsheet detailing the results of regression analysis in overlaps of predicted habitable ranges between baboon species through time.

File name: Supplementary Movie 1

Description: Papio model through time

File name: Supplementary Movie 2

Description: P. anubis model through time

File name: Supplementary Movie 3

Description: P. cynocephalus model through time

File name: Supplementary Movie 4

Description: P. hamadryas model through time

File name: Supplementary Movie 5

Description: P. kindae model through time

File name: Supplementary Movie 6

Description: P. papio model through time

File name: Supplementary Movie 7

Description: P. ursinus model through time
